# 2-(4-Iodo­phen­yl)-5-methyl-3-methyl­sulfinyl-1-benzofuran

**DOI:** 10.1107/S1600536808013706

**Published:** 2008-05-10

**Authors:** Hong Dae Choi, Pil Ja Seo, Byeng Wha Son, Uk Lee

**Affiliations:** a Department of Chemistry, Dongeui University, San 24 Kaya-dong, Busanjin-gu, Busan 614-714, Republic of Korea; b Department of Chemistry, Pukyong National University, 599-1 Daeyeon 3-dong, Nam-gu, Busan 608-737, Republic of Korea

## Abstract

The title compound, C_16_H_13_IO_2_S, was prepared by the oxidation of 2-(4-iodo­phen­yl)-5-methyl-3-methyl­sulfanyl-1-benzofuran with 3-chloro­peroxy­benzoic acid. The 4-iodo­phenyl ring makes a dihedral angle of 37.97 (9)° with the plane of the benzofuran fragment, and the O atom and the methyl group of the methyl­sulfinyl substituent lie on opposite sides of this plane. The mol­ecular packing is stabilized by C—H⋯π inter­actions between H atoms on the 4-iodo­phenyl ring and the benzofuran rings, and by an I⋯O halogen bond of 3.252 (2) Å with a nearly linear C—I⋯O angle of 163.06 (8)°. In addition, the stacked mol­ecules exhibit inversion-related S⋯O contacts [3.209 (2) Å] involving the sulfinyl groups.

## Related literature

For the crystal structures of similar 2-aryl-5-methyl-3-methyl­sulfinyl-1-benzofuran compounds, see: Choi *et al.* (2007*a* ▶,*b* ▶). For a review of halogen bonding, see: Politzer *et al.* (2007 ▶). For details of sulfin­yl–sulfinyl inter­actions, see: Choi *et al.* (2007*c* ▶). For a review of carbon­yl–carbonyl inter­actions, see: Allen *et al.* (1998 ▶).
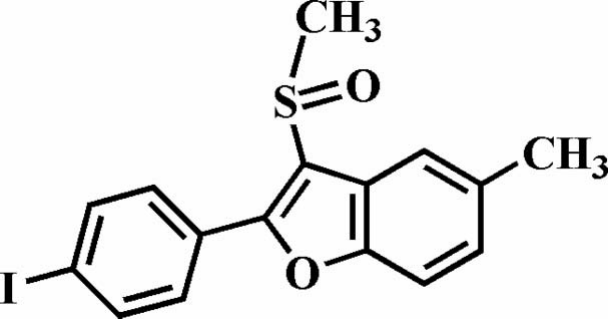


## Experimental

### 

#### Crystal data

C_16_H_13_IO_2_S
*M* *_r_* = 396.22
Monoclinic, 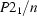

*a* = 9.258 (2) Å
*b* = 15.939 (3) Å
*c* = 10.299 (2) Å
β = 103.471 (3)°
*V* = 1477.9 (5) Å^3^
*Z* = 4
Mo *K*α radiation
μ = 2.31 mm^−1^
*T* = 173 (2) K
0.40 × 0.30 × 0.30 mm

#### Data collection

Bruker SMART CCD diffractometer
Absorption correction: multi-scan (*SADABS*; Sheldrick, 2000 ▶) *T*_min_ = 0.443, *T*_max_ = 0.508
8743 measured reflections
3227 independent reflections
2934 reflections with *I* > 2σ(*I*)
*R*_int_ = 0.030

#### Refinement

*R*[*F* ^2^ > 2σ(*F* ^2^)] = 0.026
*wR*(*F* ^2^) = 0.065
*S* = 1.15
3227 reflections
183 parameters
H-atom parameters constrained
Δρ_max_ = 0.54 e Å^−3^
Δρ_min_ = −0.94 e Å^−3^

### 

Data collection: *SMART* (Bruker, 2001 ▶); cell refinement: *SAINT* (Bruker, 2001 ▶); data reduction: *SAINT*; program(s) used to solve structure: *SHELXS97* (Sheldrick, 2008 ▶); program(s) used to refine structure: *SHELXL97* (Sheldrick, 2008 ▶); molecular graphics: *ORTEP-3* (Farrugia, 1997 ▶) and *DIAMOND* (Brandenburg, 1998 ▶); software used to prepare material for publication: *SHELXL97*.

## Supplementary Material

Crystal structure: contains datablocks I, global. DOI: 10.1107/S1600536808013706/sj2496sup1.cif

Structure factors: contains datablocks I. DOI: 10.1107/S1600536808013706/sj2496Isup2.hkl

Additional supplementary materials:  crystallographic information; 3D view; checkCIF report

## Figures and Tables

**Table 1:** Hydrogen-bond geometry (Å, °)

*D*—H⋯*A*	*D*—H	H⋯*A*	*D*⋯*A*	*D*—H⋯*A*
C10—H10⋯*Cg*1^i^	0.95	3.01	3.617 (4)	125
C11—H11⋯*Cg*2^i^	0.95	2.77	3.643 (4)	148

Symmetry code: (i) 
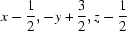
. *Cg*1 and *Cg*2 are the centroids of the C2–C7 benzene ring and the O1/C8/C1/C2/C7 furan ring, respectively.

**Fig. 1.**
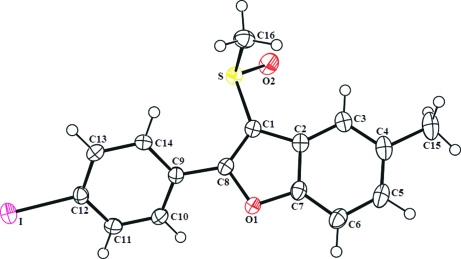
The molecular structure of the title compound, showing displacement ellipsoids drawn at the 50% probability level.

**Fig. 2.**
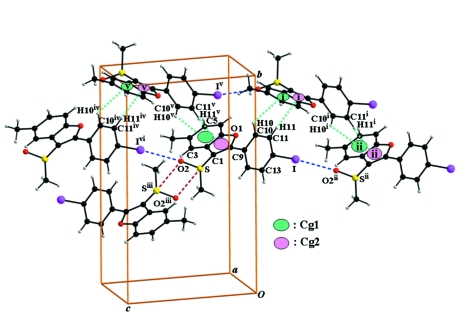
C—H···π, I···O halogen bond and S···O interactions (dotted lines) in the title compound. Cg denotes the ring centroids. [Symmetry code: (i) x - 1/2, -y + 3/2, z - 1/2; (ii) x + 1/2, y, z - 1; (iii) -x + 1, -y + 1, -z + 1; (iv) x + 1, y, z + 1; (v) x + 1/2, -y + 3/2, z + 1/2.]

## supplementary crystallographic information

### Comment

This work is related to our preceding communications on the synthesis and structure of 2-aryl-5-methyl-3-methylsulfinyl-1-benzofuran derivatives, *viz*. 2-(4-bromophenyl)-5-methyl-3-methylsulfinyl-1-benzofuran (Choi *et al.*, 2007*a*) and 2-(4-bromophenyl)-5,7-dimethyl-3-methylsulfinyl-1-benzofuran (Choi *et al.*, 2007*b*). Here we report the crystal structure of the title compound, 2-(4-iodophenyl)-5-methyl-3-methylsulfinyl-1-benzofuran (Fig. 1).

The benzofuran unit is essentially planar, with a mean deviation of 0.008Å from the least-squares plane defined by the nine constituent atoms. The 4-iodophenyl ring (C9—C14) makes a dihedral angle of 37.97 (9)° with the plane of the benzofuran fragment. The molecular packing (Fig. 2) is stabilized by two different C—H···π interactions within each stack of molecules; one between a 4-iodophenyl H atom and the benzene ring (*Cg*1^i^), with a C10—H10···*Cg*1^i^ separation of 3.617 (4) Å, and a second between a 4-iodophenyl H atom and the furan ring (*Cg*2^i^), with a C11—H11···*Cg*2^i^ separation of 3.643 (4) Å, (Fig. 2 and Table 1; *Cg*1 and *Cg*2 are the centroids of the C2—C7 benzene ring and the O1/C8/C1/C2/C7 furan ring, respectively, symmetry code as in Fig. 2). The molecular packing is further stabilized by an I···O halogen bond (Politzer *et al.*, 2007) between the iodine atom and the oxygen of a neighbouring S═O unit, with a I···O2^ii^ distance of 3.252 (2) Å (symmetry code as Fig. 2). In addition, the crystal packing exhibits a sulfinyl-sulfinyl interaction (Choi *et al.*, 2007*c*) interpreted as similar to a type-II carbonyl-carbonyl interaction (Allen *et al.*, 1998), with S···O2^iii^ and O2···S^iii^ distance of 3.209 (2)Å (symmetry code as in Fig. 2)

### Experimental

3-Chloroperoxybenzoic acid (77%, 247 mg, 1.1 mmol) was added in small portions to a stirred solution of 2-(4-iodophenyl)-5-methyl-3-methylsulanyl-1-benzofuran (380 mg, 1.0 mmol) in dichloromethane (30 ml) at 273 K. After stirring at room temperature for 2 h, the mixture was washed with saturated sodium bicarbonate solution and the organic layer was separated, dried over magnesium sulfate, filtered and concentrated in vacuum. The residue was purified by column chromatography (ethyl acetate) to afford the title compound as a colorless solid [yield 84%, m.p. 472–473 K; *R*_f_ = 0.61 (ethyl acetate)]. Single crystals suitable for X-ray diffraction were prepared by slow evaporation of a solution of title compound in tetrahydrofuran at room temperature. Spectroscopic analysis: ^1^H NMR (CDCl_3_, 400 MHz) δ 2.48 (s, 3H), 3.11 (s, 3H), 7.22 (d, J = 8.04 Hz, 1H), 7.42 (d, J = 8.44 Hz, 1H), 7.75 (d, J = 6.96 Hz, 2H), 7.85 (d, J = 6.96 Hz, 2H), 7.99 (s, 1H); EI—MS 396 [*M*^+^].

### Refinement

All H atoms were geometrically located in ideal positions and refined using a riding model, with C—H = 0.95 Å for aromatic H atoms and 0.98 Å for methyl H atoms, and with *U*_iso_(H) = 1.2Ueq(C) for aromatic H atoms, and 1.5Ueq(C) for methyl H atoms.

### Crystal data

**Table:**

C_16_H_13_IO_2_S	*F*_000_ = 776
*M**_r_* = 396.22	*D*_x_ = 1.781 Mg m^−^^3^
Monoclinic, *P*2_1_/*n*	Melting point = 472–473 K
Hall symbol: -P 2yn	Mo *K*α radiation λ = 0.71073 Å
*a* = 9.258 (2) Å	Cell parameters from 6653 reflections
*b* = 15.939 (3) Å	θ = 2.4–28.2º
*c* = 10.299 (2) Å	µ = 2.31 mm^−^^1^
β = 103.471 (3)º	*T* = 173 (2) K
*V* = 1477.9 (5) Å^3^	Block, colorless
*Z* = 4	0.40 × 0.30 × 0.30 mm

### Data collection

**Table:**

Bruker SMART CCD diffractometer	3227 independent reflections
Radiation source: fine-focus sealed tube	2934 reflections with *I* > 2σ(*I*)
Monochromator: graphite	*R*_int_ = 0.030
Detector resolution: 10.0 pixels mm^-1^	θ_max_ = 27.0º
*T* = 173(2) K	θ_min_ = 2.4º
φ and ω scans	*h* = −11→6
Absorption correction: multi-scan(SADABS; Sheldrick, 2000)	*k* = −20→20
*T*_min_ = 0.443, *T*_max_ = 0.508	*l* = −11→13
8743 measured reflections	

### Refinement

**Table:**

Refinement on *F*^2^	Secondary atom site location: difference Fourier map
Least-squares matrix: full	Hydrogen site location: inferred from neighbouring sites
*R*[*F*^2^ > 2σ(*F*^2^)] = 0.026	H-atom parameters constrained
*wR*(*F*^2^) = 0.065	*w* = 1/[σ^2^(*F*_o_^2^) + (0.0226*P*)^2^ + 1.1637*P*] where *P* = (*F*_o_^2^ + 2*F*_c_^2^)/3
*S* = 1.15	(Δ/σ)_max_ = 0.001
3227 reflections	Δρ_max_ = 0.54 e Å^−^^3^
183 parameters	Δρ_min_ = −0.94 e Å^−^^3^
Primary atom site location: structure-invariant direct methods	Extinction correction: none

### Special details

**Table:**

Geometry. All e.s.d.'s (except the e.s.d. in the dihedral angle between two l.s. planes) are estimated using the full covariance matrix. The cell e.s.d.'s are taken into account individually in the estimation of e.s.d.'s in distances, angles and torsion angles; correlations between e.s.d.'s in cell parameters are only used when they are defined by crystal symmetry. An approximate (isotropic) treatment of cell e.s.d.'s is used for estimating e.s.d.'s involving l.s. planes.
Refinement. Refinement of *F*^2^ against ALL reflections. The weighted *R*-factor *wR* and goodness of fit *S* are based on *F*^2^, conventional *R*-factors *R* are based on *F*, with *F* set to zero for negative *F*^2^. The threshold expression of *F*^2^ > σ(*F*^2^) is used only for calculating *R*-factors(gt) *etc*. and is not relevant to the choice of reflections for refinement. *R*-factors based on *F*^2^ are statistically about twice as large as those based on *F*, and *R*- factors based on ALL data will be even larger.

### Fractional atomic coordinates and isotropic or equivalent isotropic displacement parameters (Å^2^)

**Table:**

	*x*	*y*	*z*	*U*_iso_*/*U*_eq_	
I	−0.134684 (19)	0.613119 (11)	−0.281817 (17)	0.03372 (7)	
S	0.51165 (7)	0.54138 (4)	0.32703 (6)	0.02482 (13)	
O1	0.61196 (19)	0.66982 (11)	0.02820 (17)	0.0251 (4)	
O2	0.5977 (2)	0.57384 (14)	0.45895 (18)	0.0389 (5)	
C1	0.5913 (3)	0.59181 (15)	0.2070 (2)	0.0220 (5)	
C2	0.7456 (3)	0.61452 (15)	0.2246 (2)	0.0231 (5)	
C3	0.8760 (3)	0.60093 (15)	0.3218 (3)	0.0262 (5)	
H3	0.8746	0.5687	0.3991	0.031*	
C4	1.0087 (3)	0.63528 (17)	0.3040 (3)	0.0309 (6)	
C5	1.0091 (3)	0.68158 (17)	0.1884 (3)	0.0329 (6)	
H5	1.1006	0.7038	0.1771	0.039*	
C6	0.8810 (3)	0.69632 (16)	0.0895 (3)	0.0308 (6)	
H6	0.8824	0.7277	0.0114	0.037*	
C7	0.7515 (3)	0.66243 (15)	0.1120 (2)	0.0249 (5)	
C8	0.5155 (3)	0.62626 (14)	0.0892 (2)	0.0228 (5)	
C9	0.3610 (3)	0.62508 (15)	0.0136 (2)	0.0217 (5)	
C10	0.3001 (3)	0.69536 (16)	−0.0607 (3)	0.0270 (5)	
H10	0.3572	0.7453	−0.0564	0.032*	
C11	0.1575 (3)	0.69273 (16)	−0.1403 (3)	0.0284 (5)	
H11	0.1162	0.7410	−0.1893	0.034*	
C12	0.0746 (3)	0.61946 (15)	−0.1483 (2)	0.0235 (5)	
C13	0.1323 (3)	0.54923 (15)	−0.0743 (2)	0.0230 (5)	
H13	0.0746	0.4995	−0.0797	0.028*	
C14	0.2744 (3)	0.55201 (15)	0.0075 (2)	0.0218 (5)	
H14	0.3134	0.5044	0.0595	0.026*	
C15	1.1498 (3)	0.62338 (19)	0.4100 (3)	0.0417 (7)	
H15A	1.1345	0.6425	0.4962	0.063*	
H15B	1.2298	0.6561	0.3867	0.063*	
H15C	1.1769	0.5638	0.4160	0.063*	
C16	0.5769 (3)	0.43689 (17)	0.3073 (3)	0.0317 (6)	
H16A	0.6833	0.4386	0.3096	0.048*	
H16B	0.5227	0.4139	0.2216	0.048*	
H16C	0.5604	0.4013	0.3801	0.048*	

### Atomic displacement parameters (Å^2^)

**Table:**

	*U*^11^	*U*^22^	*U*^33^	*U*^12^	*U*^13^	*U*^23^
I	0.02443 (11)	0.04004 (12)	0.03256 (11)	−0.00188 (7)	−0.00175 (7)	0.00229 (7)
S	0.0250 (3)	0.0303 (3)	0.0206 (3)	−0.0021 (2)	0.0083 (2)	−0.0031 (2)
O1	0.0205 (8)	0.0272 (9)	0.0277 (9)	−0.0025 (7)	0.0057 (7)	0.0030 (7)
O2	0.0418 (12)	0.0538 (13)	0.0214 (9)	−0.0143 (10)	0.0082 (8)	−0.0077 (9)
C1	0.0224 (12)	0.0229 (11)	0.0215 (11)	−0.0001 (9)	0.0066 (9)	−0.0038 (9)
C2	0.0239 (13)	0.0225 (11)	0.0233 (11)	0.0009 (9)	0.0062 (10)	−0.0069 (9)
C3	0.0251 (13)	0.0251 (12)	0.0274 (12)	0.0031 (9)	0.0039 (10)	−0.0077 (9)
C4	0.0231 (13)	0.0290 (13)	0.0382 (14)	0.0037 (10)	0.0024 (11)	−0.0142 (11)
C5	0.0209 (13)	0.0293 (13)	0.0497 (16)	−0.0028 (10)	0.0108 (11)	−0.0095 (12)
C6	0.0257 (13)	0.0281 (13)	0.0413 (15)	−0.0005 (10)	0.0133 (11)	−0.0007 (11)
C7	0.0212 (12)	0.0227 (12)	0.0313 (12)	−0.0002 (9)	0.0067 (10)	−0.0042 (10)
C8	0.0217 (12)	0.0216 (11)	0.0259 (12)	−0.0013 (9)	0.0076 (10)	−0.0025 (9)
C9	0.0208 (12)	0.0245 (12)	0.0203 (11)	−0.0006 (9)	0.0055 (9)	−0.0019 (9)
C10	0.0252 (13)	0.0236 (12)	0.0319 (13)	−0.0040 (10)	0.0062 (10)	0.0030 (10)
C11	0.0286 (13)	0.0250 (12)	0.0308 (13)	0.0012 (10)	0.0051 (11)	0.0068 (10)
C12	0.0190 (11)	0.0295 (13)	0.0221 (11)	0.0002 (9)	0.0046 (9)	−0.0014 (9)
C13	0.0252 (12)	0.0211 (11)	0.0245 (11)	−0.0034 (9)	0.0095 (10)	−0.0024 (9)
C14	0.0247 (12)	0.0209 (11)	0.0202 (11)	0.0007 (9)	0.0062 (9)	0.0014 (9)
C15	0.0240 (14)	0.0445 (17)	0.0514 (18)	0.0020 (12)	−0.0017 (13)	−0.0162 (14)
C16	0.0351 (15)	0.0325 (14)	0.0296 (13)	0.0038 (11)	0.0116 (11)	0.0046 (11)

### Geometric parameters (Å, °)

**Table:**

I—O2^i^	3.252 (2)	C6—H6	0.9500
I—C12	2.101 (3)	C8—C9	1.461 (3)
S—O2	1.4981 (19)	C9—C10	1.399 (3)
S—O2^ii^	3.209 (2)	C9—C14	1.407 (3)
S—C1	1.773 (2)	C10—C11	1.383 (4)
S—C16	1.799 (3)	C10—H10	0.9500
O1—C7	1.381 (3)	C11—C12	1.389 (3)
O1—C8	1.391 (3)	C11—H11	0.9500
C1—C8	1.367 (3)	C12—C13	1.389 (3)
C1—C2	1.444 (3)	C13—C14	1.388 (3)
C2—C3	1.394 (4)	C13—H13	0.9500
C2—C7	1.400 (3)	C14—H14	0.9500
C3—C4	1.395 (4)	C15—H15A	0.9800
C3—H3	0.9500	C15—H15B	0.9800
C4—C5	1.402 (4)	C15—H15C	0.9800
C4—C15	1.507 (4)	C16—H16A	0.9800
C5—C6	1.391 (4)	C16—H16B	0.9800
C5—H5	0.9500	C16—H16C	0.9800
C6—C7	1.383 (4)		
			
C12—I—O2^i^	163.06 (8)	C10—C9—C8	120.1 (2)
O2—S—C1	104.84 (12)	C14—C9—C8	120.7 (2)
O2—S—C16	107.47 (13)	C11—C10—C9	120.5 (2)
C1—S—C16	97.80 (12)	C11—C10—H10	119.8
C7—O1—C8	106.30 (18)	C9—C10—H10	119.8
C8—C1—C2	107.4 (2)	C10—C11—C12	119.9 (2)
C8—C1—S	126.1 (2)	C10—C11—H11	120.1
C2—C1—S	125.89 (18)	C12—C11—H11	120.1
C3—C2—C7	119.1 (2)	C13—C12—C11	120.6 (2)
C3—C2—C1	135.8 (2)	C13—C12—I	119.88 (18)
C7—C2—C1	105.1 (2)	C11—C12—I	119.44 (18)
C2—C3—C4	119.1 (2)	C14—C13—C12	119.7 (2)
C2—C3—H3	120.5	C14—C13—H13	120.1
C4—C3—H3	120.5	C12—C13—H13	120.1
C3—C4—C5	119.7 (2)	C13—C14—C9	120.2 (2)
C3—C4—C15	119.8 (3)	C13—C14—H14	119.9
C5—C4—C15	120.6 (3)	C9—C14—H14	119.9
C6—C5—C4	122.7 (2)	C4—C15—H15A	109.5
C6—C5—H5	118.7	C4—C15—H15B	109.5
C4—C5—H5	118.7	H15A—C15—H15B	109.5
C7—C6—C5	115.9 (3)	C4—C15—H15C	109.5
C7—C6—H6	122.1	H15A—C15—H15C	109.5
C5—C6—H6	122.1	H15B—C15—H15C	109.5
O1—C7—C6	125.7 (2)	S—C16—H16A	109.5
O1—C7—C2	110.7 (2)	S—C16—H16B	109.5
C6—C7—C2	123.6 (2)	H16A—C16—H16B	109.5
C1—C8—O1	110.5 (2)	S—C16—H16C	109.5
C1—C8—C9	134.8 (2)	H16A—C16—H16C	109.5
O1—C8—C9	114.6 (2)	H16B—C16—H16C	109.5
C10—C9—C14	119.1 (2)		
			
O2—S—C1—C8	−136.9 (2)	C1—C2—C7—C6	−179.6 (2)
C16—S—C1—C8	112.7 (2)	C2—C1—C8—O1	0.4 (3)
O2—S—C1—C2	33.6 (2)	S—C1—C8—O1	172.33 (17)
C16—S—C1—C2	−76.9 (2)	C2—C1—C8—C9	178.0 (3)
C8—C1—C2—C3	178.7 (3)	S—C1—C8—C9	−10.1 (4)
S—C1—C2—C3	6.8 (4)	C7—O1—C8—C1	−0.5 (3)
C8—C1—C2—C7	−0.2 (3)	C7—O1—C8—C9	−178.63 (19)
S—C1—C2—C7	−172.09 (18)	C1—C8—C9—C10	146.5 (3)
C7—C2—C3—C4	−0.1 (3)	O1—C8—C9—C10	−36.0 (3)
C1—C2—C3—C4	−178.9 (3)	C1—C8—C9—C14	−37.5 (4)
C2—C3—C4—C5	−0.9 (4)	O1—C8—C9—C14	140.0 (2)
C2—C3—C4—C15	178.2 (2)	C14—C9—C10—C11	−0.8 (4)
C3—C4—C5—C6	1.0 (4)	C8—C9—C10—C11	175.3 (2)
C15—C4—C5—C6	−178.1 (2)	C9—C10—C11—C12	−1.0 (4)
C4—C5—C6—C7	0.1 (4)	C10—C11—C12—C13	1.7 (4)
C8—O1—C7—C6	179.8 (2)	C10—C11—C12—I	−174.85 (19)
C8—O1—C7—C2	0.4 (2)	C11—C12—C13—C14	−0.6 (4)
C5—C6—C7—O1	179.4 (2)	I—C12—C13—C14	175.94 (17)
C5—C6—C7—C2	−1.2 (4)	C12—C13—C14—C9	−1.2 (3)
C3—C2—C7—O1	−179.3 (2)	C10—C9—C14—C13	1.9 (3)
C1—C2—C7—O1	−0.2 (3)	C8—C9—C14—C13	−174.2 (2)
C3—C2—C7—C6	1.3 (4)		

Symmetry codes: (i) *x*−1, *y*, *z*−1; (ii) −*x*+1, −*y*+1, −*z*+1.

### Hydrogen-bond geometry (Å, °)

**Table:**

*D*—H···*A*	*D*—H	H···*A*	*D*···*A*	*D*—H···*A*
C10—H10···Cg1^iii^	0.95	3.01	3.617 (4)	125
C11—H11···Cg2^iii^	0.95	2.77	3.643 (4)	148

Symmetry codes: (iii) *x*−1/2, −*y*+3/2, *z*−1/2.
